# Anti-biofilm Action of *Chenopodium ambrosioides* Extract, Cytotoxic Potential and Effects on Acrylic Denture Surface

**DOI:** 10.3389/fmicb.2019.01724

**Published:** 2019-08-13

**Authors:** Patrícia Maria Wiziack Zago, Simeone Júlio dos Santos Castelo Branco, Letícia de Albuquerque Bogéa Fecury, Letícia Torres Carvalho, Cláudia Quintino Rocha, Petrus Levid Barros Madeira, Eduardo Martins de Sousa, Fabiana Suelen Figuerêdo de Siqueira, Marco Aurélio Benini Paschoal, Rafael Soares Diniz, Letícia Machado Gonçalves

**Affiliations:** ^1^Department of Medicine, São Leopoldo Mandic Faculty, Araras, Brazil; ^2^Department of Dentistry, Post-Graduate Program in Dentistry, CEUMA University, São Luís, Brazil; ^3^Department of Chemistry, Federal University of Maranhão, São Luís, Brazil; ^4^Department of Immunology and Microbiology of Respiratory Tract Infections, Post-Graduate Program in Parasite Biology, CEUMA University, São Luís, Brazil; ^5^Department of Paediatric Dentistry and Orthodontics, Federal University of Minas Gerais, Belo Horizonte, Brazil

**Keywords:** biofilms, natural products, *C. albicans*, candidosis, denture

## Abstract

Considering the challenge to control *Candida*-associated denture stomatitis, the search for antifungal substances derived from natural sources has become a trend in the literature. In this study the following effects of *Chenopodium ambrosioides* extract (CAE) were investigated: action against biofilms of *Candida albicans*, its cytotoxic potential, and changes caused in acrylic resin. The CAE was characterized by High Performance Liquid Chromatography (HPLC). The susceptibility of *C. albicans* to CAE was investigated by Minimum Inhibitory Concentration and Minimum Fungicidal Concentration (MIC and MFC) tests. Acrylic resin disks were fabricated, and *C. albicans* biofilms were developed on these for 48 h. Afterward the disks were immersed for 10 min in: PBS (Negative Control); 1% Sodium Hypochlorite (1% SH, Positive Control) or CAE at MIC or 5xMIC. The biofilms were investigated relative to counts and metabolic activity. The cytotoxic potential in keratinocytes and fibroblasts was verified by MTT test. Change in color and roughness of the acrylic resin was analyzed after 28 days of immersion in CAE. The data were analyzed by the ANOVA considering a 5% level of significance. The main compounds detected by HPLC were kaempferol and quercetin. Both MIC and MFC obtained the value of 0.25 mg/mL. The MIC was sufficient to significantly reduce the counts and activity of the biofilm cells (*p* < 0.0001), while 5xMIC resulted in almost complete eradication, similar to 1% SH. Keratinocytes and fibroblasts exposed to the MIC and 5xMIC presented cell viability similar to that of the Control Group (*p* > 0.05). No important changes in acrylic resin color and roughness were detected, even after 28 days. It could be concluded that the immersion of acrylic resin in *C. ambrosioides* extract in its minimum inhibitory concentration was effective for the reduction of *C. albicans* biofilms without any evidence of cytotoxic effects or changes in roughness and color of this substrate.

## Introduction

With the purpose of re-establishing the function, esthetics and oral health of partially or completely edentulous patients, removable dental prostheses fabricated of poly (methyl methacrylate) acrylic resin (PMMA) are an excellent treatment option (Gad et al., 2017). However, after insertion in the oral cavity, the prostheses are exposed to diverse microorganisms that may adhere to their surface, and if this process is not interrupted by means of adequate cleaning or treatment with antifungal agents, this will result in the development of microbial communities denominated biofilm (Hannah et al., 2017). Accumulation of this biofilm works as a reservoir of microorganism with the potential to cause infections such as *Candida-*associated denture stomatitis (CADS), with *Candida albicans* being the main microorganism involved in its etiology (Gleiznys et al., 2015).

The most popular method for controlling biofilm on dentures is brushing with toothpaste, as it is easily accessible and low cost (Pellizzaro et al., 2012). However, there are pores in acrylic resin, and in some situation, the toothbrush bristles are unable to clean these pores adequately (Paranhos Hde et al., 2013). Added to this, many dental prostheses wearers are known to be elderly patients, who may have diminished visual acuity or even loss of motor ability, thus compromising the performance of adequate cleaning (Padilha et al., 2007).

Immersion in chemical disinfectant or cleaning solutions has been suggested as an important auxiliary method for eliminating biofilm (Gantait et al., 2016). Among the solutions, sodium hypochlorite is widely used, as it is capable of performing efficient cleaning (Sousa-Porta et al., 2013; Arruda et al., 2018). However, it has a disagreeable taste and smell, in addition to change the roughness and color of acrylic resin when used for a prolonged period of time (Sousa-Porta et al., 2013; Salloum, 2014; Arruda et al., 2018). Usually, the chemical cleaners are commercially available substances, whose cleaning action occurs through the mechanical effect of effervescence produced when the product is dissolved in water, resulting in an alkaline solution of hydrogen peroxide (Jose et al., 2010). Various studies have investigated the effect of different cleaners on biofilm formed on dental prosthesis surfaces (Lucena-Ferreira et al., 2013, 2014), however, this method has been observed to fail to remove biofilms of *C. albicans* (Jose et al., 2010; Lucena-Ferreira et al., 2013, 2014).

In situations in which CADS has become a persistent or recurrent infection, studies have recommended the prescription of antifungal agents, among which the most outstanding are the group of polyenes and azoles (i.e., nystatin and fluconazole, respectively) (Di Stasio et al., 2018). The indiscriminate use of these antifungal agents appears to have been a preponderant factor for the selection of resistant strains of *C. albicans* (Niimi et al., 2010; Taff et al., 2013). Furthermore, the complex structure of biofilm formed appears to make their diffusion difficult, and consequently, limit the action of the antifungal agents (Taff et al., 2013).

The limitations perceived in biofilm control offered by chemical/mechanical methods have important implications in the failure of treatment for CADS. Thus, the search for antifungal substances obtained from alternative sources has become a trend in the medical-dental literature (Nett, 2014; Vieira et al., 2014; Jesus et al., 2018). Considering that the majority of existent antifungal agents on the market are of synthetic origin, the interest for natural inputs has regained attention in the search for active principles for the formulation of effective products with low toxicity for the control of infections (Duarte et al., 2005; Rios and Recio, 2005). Diminishing the treatment costs of infections and favoring the population with new options of therapy is a great challenge to science.

Among the natural inputs, plants used in popular medicine represent a promising option for the prevention and treatment of fungal infections (Vieira et al., 2014; Madeira et al., 2016; Gonçalves et al., 2018). Among these plants, *Chenopodium ambrosioides* – popularly known as “mastruz” or “Erva de Santa Maria” has attracted the attention of researchers (Jardim et al., 2010; Shah and Khan, 2017; Jesus et al., 2018). Mastruz is a native plant of Central and South America, and it is widely distributed throughout Brazilian territory. There are reports of its use in popular medicine for the treatment of wounds, vaginal discharge, inflammatory processes, antihelmintic agent and oral antiseptic treatment (De Queiroz et al., 2014; Bieski et al., 2015). Previous studies have demonstrated its medicinal potential against the action of endoparasites and fungi (Kumar et al., 2007; Jesus et al., 2018), among these, some species of *Candida* (Chekem et al., 2010; Jardim et al., 2010). Moreover, anti-leishmania (De Queiroz et al., 2014), anxiolytic, healing, anti-inflammatory (Trivellato Grassi et al., 2013; Penha et al., 2017) and antioxidant (Kumar et al., 2007) activities have been described.

From its antimicrobial effect (Kumar et al., 2007; Chekem et al., 2010; Jardim et al., 2010; Jesus et al., 2018), and anti-inflammatory property (Trivellato Grassi et al., 2013; Penha et al., 2017) the potential of mastruz could be inferred as an auxiliary method in the control of CADS and/or prevention of biofilm on the surface of removable dental prostheses. However, in the process of selection and indication of a substance for the treatment of candidosis, the effect of the substance not only on biofilm and cells of the host, but also on the prosthesis substrate must be considered; that is, the effects on acrylic resin (Madeira et al., 2016; Arruda et al., 2018). Among the main disadvantages of using auxiliary chemical substances for biofilm control, there are known to be the changes that these may induce in the color and roughness of acrylic resin (Sousa-Porta et al., 2013; Madeira et al., 2016). Modulation of the host immune response through the anti-inflammatory activity, reduction in biofilm, and absence of harmful effects on acrylic resin would justify the therapeutic use of mastruz in patients with candidosis, and in future, the development of a product that would be easily accessible to the population.

Therefore, the aim of this study was to investigate the *in vitro* effect of the *C. ambrosioides* extract on *C. albicans* biofilms, cytotoxic potential and possible changes in the color and roughness acrylic resin.

## Materials and Methods

### Experimental Design

The hydro alcoholic *C. ambrosioides* extract (CAE) was prepared, and characterized by High Performance Liquid Chromatography (HPLC). The susceptibility of planktonic cells of *C. albicans* (ATCC 90028) to CAE was investigated by means of the Minimum Inhibitory Concentration (MIC) and Minimum Fungicidal Concentration (MFC) tests. For analysis of the biofilms, disks of PMMA were fabricated in accordance with the manufacturer’s instructions, and their surface roughness was standardized. Afterward, biofilms of *C. albicans* were developed on these disks for 48 h. The biofilms were immersed for 10 min in the following treatments: Phosphate-Buffered Saline (PBS, Negative Control), 1% Sodium Hypochlorite (1% SH Positive Control) or CAE in the concentrations of MIC or 5xMIC. Subsequently the biofilms were investigated relative to cell counts and metabolic activity. The cytotoxic potential in keratinocytes and fibroblasts was verified by means of the MTT test. Cells experiments were assembled in triplicate and repeated at three different moments. Considering the effects on the acrylic resin substrate, these were measured by means of the surface roughness and color change tests after 28 days of immersion in different concentrations of CAE. Exposure to distilled water and 1% SH was used as controls. All experiments were performed in triplicate, in the different time intervals.

### Collection, Botanical Identification, and Preparation of the Extract

The *C. ambrosioides* leaves were cultivated in an experimental field of the Federal University of Maranhão, São Luís, Brazil. The sample was collected from June 2017 to November 2017. The exsiccata were prepared and sent to the “Herbário Ático Seabra” of the Federal University of Maranhão for botanical identification. The leaves were separately dried in an air circulating oven at 37°C for 48 h, followed by grinding in a mill. The dry ground material (approximately 200 g) was macerated with 70% ethanol for 24 h, at ambient temperature. This process was repeated four times, and the extract obtained was filtered and then concentrated by using a rotary evaporator to remove the ethanol. The dry residue was diluted in distilled water up to a final concentration of 100 mg/mL, filtered through a membrane of 0.22 μg/mL and stored in amber flasks until the experiments were performed.

### Characterization of the Extract

The chemical constituents of the CAE were analyzed by HPLC by using an LC-10AD (Shimadzu, Japan) system equipped with a diode-array detector (DAD) and coupled to a mass spectrometer Esquire 3000 Plus (Bruker Daltonics, Bremen, Germany) using electrospray ionization (ESI). Separation was performed with a Phenomenex Kinetex C-18 column (250 × 4.6 mm, 5 μm; Torrance, CA, United States). Phosphoric acid was used as the A mobile phase and methanol as the B mobile phase. The elution gradient was prepared as follows: 5–20 min from 55 to 10% A, and afterward for 20–30 min from 10 to 0% A. The injection volume consisted of 20 μL of the reconstituted sample and flow rate of 1 mL/min. Detection was performed by a DAD at 470 nm and a direct mass spectrometry method (ESI) with the voltage maintained at 4.0 kV, ion source of 40 V and capillary temperature of 320°C. The compounds were identified based on the retention time and molecular mass.

### Susceptibility Tests

To evaluate the susceptibility of planktonic cells to the CAE, MIC, and MFC analyses were performed. As the control group a fluconazole solution was prepared, the action of which has been well established in the literature (Madeira et al., 2016; Di Stasio et al., 2018). For reactivation of the microorganism and preparation of the inoculum, a reference strain of *C. albicans* (ATCC 90028) was used. The strain was reactivated from its original culture on Sabouraud Dextrose Agar (SDA) plates at 37°C, for 24 h. To prepare the *C. albicans* inoculum, colonies were suspended in Yeast Nitrogen Base (YNB) broth enriched with 50 mM of glucose. The set was incubated for 20 h, at 37°C and was then centrifuged (5000 rpm, 4°C) and washed with PBS. An aliquot of centrifuged cells was transferred to a tube containing saline solution and the turbidity of this content was adjusted with a spectrophotometer, assuring a suspension of ≈10^7^ cells/mL.

The MIC was determined by the micro dilution method in broth recommended by the Clinical Laboratory Standards Institute (CLSI, 2008). From the initial concentration of the CAE, which was defined in pilot tests, serial dilution were made in 96-well plates. The wells containing the different dilutions of the extracts, controls (positive and negative) and the inoculum were incubated at 37°C for 48 h. Readout of the test was by visual comparison, and the MIC corresponded to the lowest concentration that prevented visible planktonic cell growth. Each concentration of the previous test, which did not show visible growth, was inoculated on an SDA plate. After 24 h of incubation at 37°C, the MFC readouts were made based on the growth of the controls, with the MFC being considered the lowest concentration of the extract that prevented fungal growth (≥99.9%).

### Preparation of Acrylic Resin Disks

For this study, 72 disks (10 mm in diameter × 2 mm thick) of PMMA acrylic resin (QC-20, Dentsply Ind e Com Ltda.) were fabricated with the use of a stainless steel muffle containing orifices in these dimensions, in accordance with the manufacturer’s instructions. After this, the disks were submitted to finishing with water abrasive papers No. 320, 400, and 600 in a horizontal polishing machine (Arotec; São Carlos, Brazil). Subsequently, they were stored in distilled water at 37°C for 48 h to eliminate the residual monomers. For standardization of the surface roughness, this was measured with a rugosimeter (Surfcorder SE 1700; Mitutoyo) with an active tip diameter of 2 μm, precision of 0.01 μm and speed of 0.5 mm/s under pressure of 0.07 N. Three measurements were performed and the arithmetic mean was calculated, thus defining the roughness value. For standardization, the variation of plus or minus 5% of the mean value was used. After standardizing the roughness, the disks were disinfected by immersion in 1% SH under constant agitation for 3 min, and then washed with distilled water for 10 min.

### Biofilm Development and Analysis

For biofilm formation, 36 acrylic resin disks were placed in a 24-well plate and exposed to the previously adjusted inoculum of *C. albicans*. The plates were incubated under constant agitation, at 37°C, for 90 min, a length of time corresponding to the cellular adhesion phase. Afterward, the disks were transferred to wells containing YNB medium supplemented with 100 mM of glucose and maintained at 37°C for 24 h to allow biofilm development. This process was repeated until the biofilm completed 48 h of existence. On conclusion of 48 h, the biofilms were immersed for 10 min in the following treatments: PBS (Negative Control), 1% SH (Positive Control), or CAE in the concentrations of MIC or 5xMIC.

Cell counts were made by means of (decimal) serial dilution. For this purpose, the biofilms developed on the disks were sonicated (7 W, 30 s) in saline solution for cell disaggregation. The suspension obtained was submitted to (decimal) serial dilution, and the product of each dilution was inoculated on SDA plates in triplicate. The plates were incubated at 37°C for 24 h. The colonies were visually quantified and the result was expressed in cells/mL.

The metabolic activity was determined by colorimetric analysis of the metabolic reduction of 2,3-bis(2-methoxy-4-nitro-5- sulfophenyl)-5-[(phenylamino)carbonyl]-2H-tetrazolium hydroxide (XTT). For this purpose the biofilms were exposed to the XTT solution and incubated at 37°C for 3 h. Afterward the content of each well was transferred and centrifuged (10,000 g, 5 min). XTT reduction was determined by the absorbance recorded in the spectrophotometer (492 nm).

### Cytotoxicity Activity

The immortalized lineages of human keratinocytes HaCaT (CLS – Cell Lines Service, Number 300493) and murine fibroblasts NIH-3T3 (ATCC^®^ CRL-1658), were kept at 37°C and 5% CO_2_ in a humid atmosphere, and cultivated in Dulbecco’s Modified Eagle Medium (DMEM; Gibco®) culture medium, enriched with 10% Fetal Bovine Serum (FBS), 100 U mL^–1^ of penicillin and 100 μg mL^–1^ of streptomycin.

The procedure adopted was developed based on the literature (Jorge et al., 2008). Basically, the test consisted of inoculating the keratinocytes (100 μL/well, 4 × 10^4^ cells mL^–1^) and fibroblasts (100 μL well^–1^; 3 × 10^4^ cells mL^–1^) in 96-well plates, and incubation (37°C, 5% CO_2_, humid atmosphere) for 24 h (period of cellular adhesion). Afterward the culture medium was removed and the cells were exposed to CAE in the concentrations of MIC, 5xMIC and 10xMIC for 24 and 48 h. Wells containing cells without CAE were used as control. Cell growth and viability were evaluated by means of the colorimetric MTT [3-(4,5-dimethylthiazol-2-yl)-2,5-diphenyltetrazolium bromide] method. For this purpose, all of the culture medium was removed and the cells were washed with saline solution for introduction of the new culture medium DMEM/FBS with the addition of MTT (0.5%). The plates were incubated for 4 h (37°C, humid atmosphere, 5% de CO_2_) protected from light. After this period, the medium was removed and 100% dimethylsulfoxide (DMSO) was added to solubilize the coloring agent that allowed optical readout at 550 nm. From the absorbance values, the percentages of cell viability were calculated, considering: %Viability = TA/T1 × 100, where TA was the mean absorbance of the treated cell (absorbance of white of the sample) and T1 the absorbance of the cellular suspension without treatment.

### Effects on Acrylic Resin

For the tests to color perception and surface roughness analysis 36 disks were immersed in distilled water (Negative Control), 1% SH (Positive Control) or CAE at MIC or 5xMIC. The disks were incubated at 37°C for 28 days and tested after time intervals of 0, 7, 14, 21, and 28 days of immersion, afterward the disks were washed in distilled water and dried with absorbent paper. The immersion medium was changed on a daily basis.

For color perception, the disks were placed on a silicone mold with an orifice, with the purpose of adapting them to a portable spectrophotometer (EasyShade Advance 4.0; Wilcos, Germany). This mold was used with the purpose of providing precise repositioning and measurement of the color of the disk surface. The color measurements were made with the use of the system CIEL^*^a ^*^b^*^. The color change (ΔE) was calculated using the following formula: ΔE^*^ = [(ΔL^*^)^2^ + (Δa^*^)^2^ + (Δb^*^)^2^]. Values of ΔE < 3.7 were considered clinically imperceptible (Moon et al., 2014).

For the surface roughness analysis, on each disk three readouts were made with a rugosimeter (Surfcorder SE 1700; Mitutoyo). The roughness of each disk was calculated by the arithmetic mean (μm). The change in surface roughness (ΔRa) was obtained by the difference between the roughness values after immersion and the initial roughness values (baseline).

### Statistical Analysis

The results were statistically analyzed by the software SAS/LAB (SAS Software, version 9.0; SAS Institute Inc., Cary, NC, United States). The normal distribution of the data was previously adjusted, and when necessary, the data were transformed as suggested by the software. The data with reference to cell counts, metabolic activity and viability of keratinocytes and fibroblasts were analyzed by the one-way ANOVA test, followed by the Tukey test, in which treatment by immersion was treated as the study factor. The data of change in color and surface roughness were analyzed by two-way ANOVA for repeated measures, followed by the Tukey test, in which the time intervals of treatment and type of immersion were considered study factors. Three measurements were performed for each sample at the same position, and an arithmetic mean value was calculated. The level of significance was 5% for all the tests.

## Results

### Characterization of the Extract

The chromatogram of CAE obtained by HPLC showed the presence of important metabolites with the flavonoids. It was possible to identify 16 of these components based on their retention times and molecular mass patterns. The compounds detected in greater abundance were kaempferol and quercetin, as described in Table 1.

**TABLE 1:** Description of compounds obtained after chemical characterization of *C. ambrosioides* by HPLC.

	**[M-H]**	**MS^*n*^**	**Compound**
1	285	257	Kaempferol
2	313	295	3′,4′-dimethoxyluteolin
3	363	295; 277; 171	NI^*a*^
4	387	163	*p*-coumaroyl acid derivative
5	391	387;163	*p*-coumaroyl acid derivative
6	295	162	NI^*a*^
7	301	285; 267; 241; 173	Hesperetin
8	431	385; 285; 152	Kaempferol 3-*O*-alpha-l-rhamnoside
9	433	387; 295	Narirutin
10	463	301	Quercetin-3-glucoside
11	495	431; 343	Digalloyl quinic acid
12	499	431; 421; 385; 285	3,4-O-(E)-*p*-coumaroylcaffeoylquinic acid
13	531	417; 285	NI^*a*^
14	563	431; 285; 151	Kaempferol-*O*-rhamnoside-pentoside
15	577	431;285	Kaempferol dirhamnoside-*O*-pentoside
16	579	447; 301	Naringin
17	593	285	Kaempferol-3-glucoside-3″-rhamnoside
18	595	301; 151	Quercetina-3-*O*-arabinoglucoside
19	613	591	NI^*a*^
20	623	447; 301	Quercetin (acyl) glucoronide-o-rhaminoside
21	695	593; 549; 430; 285	NI^*a*^

*^*a*^Not identified.*

### Susceptibility Tests

Both the MIC and MFC of CAE observed for planktonic cells of *C. albicans* obtained the value of 0.25 mg/mL, demonstrating a fungicidal pattern of behavior. The fluconazol values were 0.5 and > 64 μg/mL for MIC and MFC, respectively.

### Biofilm Analyses

**FIGURE 1:**
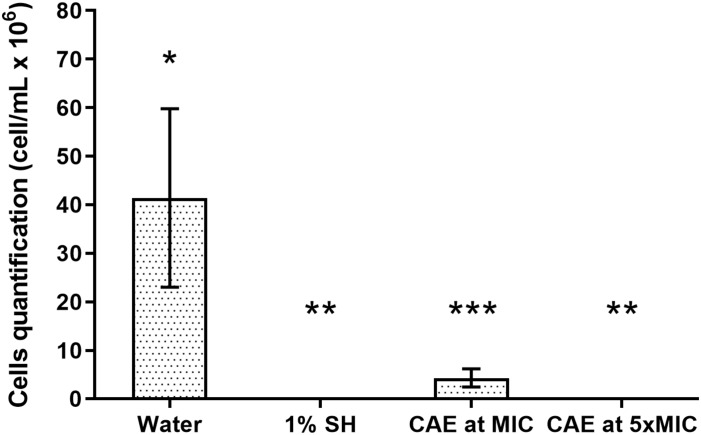
Effect of CAE on cell counts of *C. albicans* biofilm. Different symbols (^*^, ^∗∗^, ^∗∗∗^) indicate statistically significant difference among the groups (one-way ANOVA followed by the Tukey test, *p* < 0.05).

**FIGURE 2:**
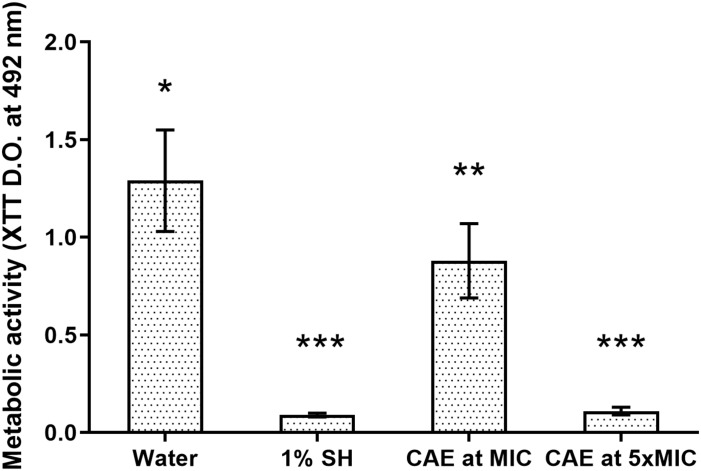
Effect of CAE on metabolic activity of *C. albicans* biofilm. Different symbols (^*^, ^∗∗^, ^∗∗∗^) indicate statistically significant difference among the groups (one-way ANOVA followed by the Tukey test, *p* < 0.05).

For the *C. albicans* biofilms, exposure to CAE for 10 min reduced significantly the cell count when compared with the Negative Control (*p* = 0.001; Figure 1). The effect of CAE at MIC was sufficient to reduce approximately 80% of the biofilm cells (*p* < 0.0001), while at 5xMIC it reached almost complete eradication of the biofilm (>99%), similar to the result obtained with 1% SH (Positive Control, *p* > 0.05). Considering the results of metabolic activity, difference was also observed between the Negative Control and CAE at MIC (*p* > 0.05), and at 5xMIC this activity was reduced to levels very close to those of 1% SH (*p* = 0.001; Figure 2).

### Cytotoxicity Activity

**FIGURE 3:**
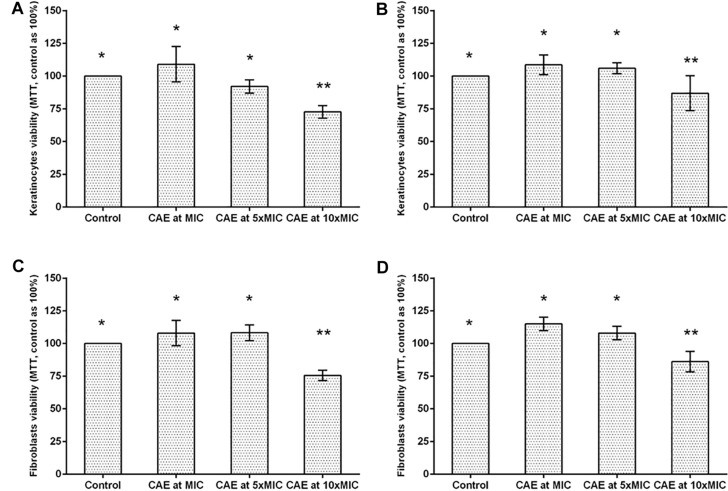
Cytotoxic activity of CAE in different concentrations on keratinocytes after **(A)** 24 h and **(B)** 48 h of exposure. Cytotoxic activity of CAE in different concentrations on fibroblasts after **(C)** 24 h and **(D)** 48 h of exposure. Different symbols (^*^, ^∗∗^) indicate statistically significant difference among the groups (one-way ANOVA followed by the Tukey test, *p* < 0.05).

Both during the period of 24 h, and after 48 h, keratinocytes and fibroblasts exposed to CAE in the concentrations of MIC and 5xMIC showed cell viability similar to that of the Control, with no statistical difference between them (*p* > 0.05). Exposure to CAE at the concentration of 10xMIC was capable of significantly reducing the viability of both cell types when compared with the Control Group (*p* < 0.05; Figure 3).

### Effects on Acrylic Resin

Considering the color change values (ΔE), when compared with the distilled water group, statistical differences were observed for immersions in both 1% SH and CAE, in all the immersion times (*p* < 0.05). In spite of this variation, all the ΔE values obtained in the immersions in CAE were <3.7, and these were therefore classified as being clinically imperceptible (Moon et al., 2014). Whereas, clinically perceptible changes were detected in the 1% SH group from the 21st day of immersion (*p* < 0.05; Table 2). As regards the surface roughness values, there was no statistically significant difference between the group with distilled water and that with CAE at MIC or 5xMIC (*p* > 0.05; Table 3). Significant changes in roughness were detected only in 1% SH group after 28 days of immersion (*p* < 0.05).

**TABLE 2:** Change in color (ΔE) of acrylic resin after immersion in CAE in different time intervals (mean ± SD, *n* = 9).

**Group**	**7 days**	**14 days**	**21 days**	**28 days**
Distilled water	1.09 ± 0.33 (A,a)	1.45 ± 0.27 (A,a)	1.54 ± 0.51 (A,a)	1.60 ± 0.24 (A,a)
1% SH	3.12 ± 0.52 (B,a)	3.22 ± 0.87 (B,a)	3.89 ± 0.58 (B,b)	4.05 ± 0.99 (B,b)
CAE at MIC	2.46 ± 0.38 (C,a)	2.41 ± 0.56 (C,a)	2.53 ± 0.69 (C,a)	2.79 ± 0.76 (C,a)
CAE at 5xMIC	2.42 ± 0.45 (C,a)	2.70 ± 0.61 (C,a)	2.88 ± 0.77 (C,a)	3.13 ± 0.57 (C,b)

*Different uppercase letters represent statistically significant differences among the groups in the same immersion period. Different lowercase letters indicate statistically significant differences between the different immersion times in the same group (ANOVA for repeated measures followed by the Tukey test, *p* < 0.05).*

**TABLE 3:** Change in surface roughness (ΔRa) of acrylic resin after immersion in CAE in different time intervals (mean ± SD, *n* = 9).

**Group**	**7 days**	**14 days**	**21 days**	**28 days**
Distilled water	0.09 ± 0.01 (A,a)	0.11 ± 0.03 (A,a)	0.11 ± 0.05 (A,a)	0.11 ± 0.04 (A,a)
1% SH	0.10 ± 0.03 (A,a)	0.14 ± 0.09 (A,a)	0.25 ± 0.06 (B,b)	0.27 ± 0.07 (B,b)
CAE at MIC	0.09 ± 0.03 (A,a)	0.10 ± 0.04 (A,a)	0.11 ± 0.03 (A,a)	0.11 ± 0.05 (A,a)
CAE at 5xMIC	0.09 ± 0.02 (A,a)	0.13 ± 0.05 (A,a)	0.12 ± 0.07 (A,a)	0.12 ± 0.09 (A,a)

*Different uppercase letters represent statistically significant differences among the groups in the same immersion period. Different lowercase letters indicate statistically significant differences between the different immersion times in the same group (ANOVA for repeated measures followed by the Tukey test, *p* < 0.05).*

## Discussion

The plants used in popular medicine are known to represent a promising option for the prevention and treatment of fungal infections (Lou et al., 2013, 2014; Vieira et al., 2014; Madeira et al., 2016; Gonçalves et al., 2018; Zhang et al., 2019). In 2009, the Brazilian Ministry of Health drew up a National List of Medicinal Plants of Interest to the Health System (“Lista Nacional de Plantas Medicinais de Interesse no Sistema de Saúde”) (RENISUS) that encourages the research of medicinal plants widely used by the Brazilian population, and *C. ambrosioides* L. is included in this list (Ministério da Saúde Organização Mundial da Saúde, 2009), thus motivating the identification of its compounds and their biological activities. In this study, for the first time in the literature, the effect of *C. ambrosioides* extract was investigated in biofilms of *C. albicans* developed on acrylic resin, simulating a condition of biofilm on the surface of a removable dental prosthesis. In an endeavor to evaluate the potential of this plant as an auxiliary method in the control of CADS, the anti-biofilm effect, toxic effect in keratinocytes and fibroblasts, and the possible deleterious effects that CAE might cause on the acrylic resin substrate were evaluated.

By means of the chemical characterization of the CAE it was possible to identify great abundance of the compounds kaempferol and quercetin – compounds belonging to the group of flavonoids (Gupta and Huang, 2014), similar to the descriptions previously given in the literature (Nedialkova et al., 2009; Jesus et al., 2018). Flavonoids have diverse pharmacological activities, among these antimicrobial and antioxidant potential. These compounds are related to inactivation of enzymes responsible for cell adhesion, which probably functions as their mechanism of antimicrobial action (Silva et al., 2012). Furthermore flavonoids are known to have potent antioxidant action, which explains their capacity to regulate the immune system (Vezza et al., 2016). Quercetin and kaempferol have previously had their antimicrobial activity attested against *Staphylococcus aureus, Escherichia coli, Enterococcus faecalis, Pseudomonas aeruginosa, Ptroteus mirabilis*, and *C. albicans* (Rashed and Butnariu, 2005; Singh et al., 2016).

As a starting point for the tests against *C. albicans*, the susceptibility of planktonic cells to CAE was investigated. By means of this test, the coincident values of MIC and MFC were observed (i.e., 0.25 mg/mL or 250 μg/mL), showing evidence of the fungicidal potential of the extract. Considering the MIC values, there is a classification in the literature for the antimicrobial activity of natural products that considers: strong inhibitors for MIC values < 100 μg/mL, moderate inhibitors for values between 100 and 500 μg/mL, weak inhibitors for values between 500 and 1000 μg/mL, and absence of activity for values > 1000 μg/mL (Holetz et al., 2002). Therefore, according to this criterion, CAE could be considered a moderate inhibitor, which partly justifies the good results obtained in the other tests performed. Moreover, the MIC value found in this study, corroborated the findings in the literature (Chekem et al., 2010; Jesus et al., 2018).

Important to point out is that the MIC value was obtained in tests that evaluated *C. albicans* in its planktonic form. In the oral cavity, especially on the surface of dentures, these cells are organized in biofilms, which, due to their structural complexity, are relatively more resistant to the action of antifungal agents (Taff et al., 2013). In the present study, it is important to point out that, the antifungal effect of the CAE was evaluated in mature biofilms of *C. albicans* (i.e., 48 h of development), and the immersion time of 10 min was chosen considering the time of immersion recommended for the positive control, 1% SH (Sousa-Porta et al., 2013; Arruda et al., 2018).

For the biofilm of *C. albicans*, it was possible to observe that 10 min of immersion in the CAE at MIC was sufficient to reduce the number of biofilm cells when compared with the negative control group. These results could be attributed to the presence of quercetin and kaempferol in its chemical composition, which have recognized antifungal potential (Silva et al., 2012; Tatsimo et al., 2012; Pinho et al., 2014; Alavarce et al., 2015; Almeida et al., 2018). In addition to interfering directly in the first mechanism of virulence of the fungus that is cell adhesion (Silva et al., 2012), these compounds are also able to prevent protein transport and cause fungal cell rupture (Alavarce et al., 2015). The reduction in metabolic activity of the biofilms exposed to the CAE, as demonstrated in the XTT test, could be attributed to being a direct consequence of reduction in the number of viable cells, as these reductions generally follow a proportional pattern (Silva et al., 2010).

A large reduction in fungal viability and activity was observed in the biofilms exposed to the CAE at 5xMIC, showing a fungicidal tendency similar to that found in the group with 1% SH. These results may have been a consequence of cell disaggregation and inhibition of the capacity of adherence. Furthermore, it is possible that the reduction in metabolic activity may have occurred due to intracellular stress arising from the action of the extract at a higher concentration (Almeida et al., 2018).

Considering that the removable dental prosthesis remains in intimate contact with the oral epithelial cells, it is of the utmost importance to evaluate the possible toxic effects of the CAE in these types of cells, guaranteeing safety in future clinical studies. According to International Standard Organization (International Standard Organization [ISO], 2009), concentrations that maintain cell viability of lower than 70% may be considered cytotoxic. In this study, it was observed that even after 48 h of exposure, keratinocytes and fibroblasts exposed to the CAE at MIC and 5xMIC showed cell viability similar to that of the control group with PBS, a buffer known to be atoxic in human cells because of its capacity for osmoregulation. The concentration of 10xMIC was observed to show a reduction in cell viability at levels considered toxic, and therefore, this concentration was excluded from the other tests used in this study. Thus, according to these results, the concentrations tested showed antifungal potential without resulting in significant damage to the host cells.

Studies have demonstrated that the main disadvantages of using auxiliary chemical substances for biofilm control were changes that these agents might induce in the color and roughness of acrylic resin, which are directly related to the longevity and esthetics of dental prostheses (Paranhos Hde et al., 2013; Sousa-Porta et al., 2013; Madeira et al., 2016).

In this study, the change in color was investigated by the colorimetric system CIEL^*^a^*^b^*^, a uniform three-dimensional system that has been widely used for determining the chromatic differences by translating their combinations into mathematical data (Sousa-Porta et al., 2013; Madeira et al., 2016). In this system, the change in color (ΔE) is defined as the relative change in color between repeated color evaluations. Considering that the values of ΔE < 3.7 are considered clinically imperceptible (Moon et al., 2014), the CAE did not cause any significant change in color in the acrylic resin even after 28 days of immersion.

In a similar manner, the CAE induced no significant changes in roughness, when compared with the negative control group in distilled water. Knowing that roughness is a crucial factor for the adhesion of microorganisms on the acrylic surface, it is of the utmost importance that chemical solutions do not change this property, because rough surfaces favor the formation of biofilms (Paranhos Hde et al., 2013). Among the solutions for chemical biofilm control, the 1% SH used in an immersion protocol for 10 min has been considered the most efficient (Lima et al., 2007; Rossato et al., 2011; Sousa-Porta et al., 2013). In spite of these good results, when used in a prolonged regimen, it is capable of degrading the acrylic resin matrix, causing a “lightening” effect and increasing the surface roughness (Lima et al., 2007), as confirmed in the present study.

Although 28 days of immersion of the resin in the CAE appeared to be a short period of time in comparison with the useful life of a dental prosthesis, the constant exposure to a solution that was changed every day was understood to be capable of significantly aging the acrylic matrix. It is known that, the daily solution exchange is a fast and reliable *in vitro* aging method to observe the degradation the of polymer by swelling the network and reducing the frictional forces between the polymer chains in a manner that ultimately weakens bonds releasing polymersin the course of days (Madeira et al., 2016).

## Conclusion

Immersion of acrylic resin for dental prostheses in the *C. ambrosioides* extract (CAE) in its MIC was effective for the reduction of *C. albicans* biofilms without any evidence of cytotoxic effects or changes in roughness and color of this substrate. In conjunction, the results of the present study suggested that *C. ambrosioides* is a possible source of bioactive substances that may be used in the future in the formulation of products that help with the control of CADS.

## Data Availability

The raw data supporting the conclusions of this manuscript will be made available by the authors, without undue reservation, to any qualified researcher.

## Author Contributions

All authors contributed to the conception or design of the study, acquisition, analysis or interpretation of the data, critically revised the manuscript, gave final approval for the manuscript, and agreed to be accountable for all aspects of the work ensuring integrity and accuracy. PZ, MP, ES, and LG drafted the manuscript.

## Conflict of Interest Statement

The authors declare that the research was conducted in the absence of any commercial or financial relationships that could be construed as a potential conflict of interest.
